# Evolutionary Pareto-optimization of stably folding peptides

**DOI:** 10.1186/1471-2105-9-109

**Published:** 2008-02-19

**Authors:** Wolfram Gronwald, Tim Hohm, Daniel Hoffmann

**Affiliations:** 1 Institute for Functional Genomics, University of Regensburg, Josef-Engert-Strasse 9, 93053 Regensburg, Germany; 2 Computer Engineering and Networks Laboratory, ETH Zurich, Gloriastrasse 35, Zürich, Switzerland; 3 Centre for Medical Biotechnology, University of Duisburg-Essen, Universitätsstrasse, 45117 Essen, Germany

## Abstract

**Background:**

As a rule, peptides are more flexible and unstructured than proteins with their substantial stabilizing hydrophobic cores. Nevertheless, a few stably folding peptides have been discovered. This raises the question whether there may be more such peptides that are unknown as yet. These molecules could be helpful in basic research and medicine.

**Results:**

As a method to explore the space of conformationally stable peptides, we have developed an evolutionary algorithm that allows optimization of sequences with respect to several criteria simultaneously, for instance stability, accessibility of arbitrary parts of the peptide, etc. In a proof-of-concept experiment we have perturbed the sequence of the peptide Villin Headpiece, known to be stable in vitro. Starting from the perturbed sequence we applied our algorithm to optimize peptide stability and accessibility of a loop. Unexpectedly, two clusters of sequences were generated in this way that, according to our criteria, should form structures with higher stability than the wild-type. The structures in one of the clusters possess a fold that markedly differs from the native fold of Villin Headpiece. One of the mutants predicted to be stable was selected for synthesis, its molecular 3D-structure was characterized by nuclear magnetic resonance spectroscopy, and its stability was measured by circular dichroism. Predicted structure and stability were in good agreement with experiment. Eight other sequences and structures, including five with a non-native fold are provided as bona fide predictions.

**Conclusion:**

The results suggest that much more conformationally stable peptides may exist than are known so far, and that small fold classes could comprise well-separated sub-folds.

## Background

Until a few years ago it was widely believed that peptides of chain lengths well below 50 residues are conformationally highly flexible or even disordered, whereas the longer polypeptide chains of proteins form stable native conformations ([1], p. 189; [2], p. 95). The latter seemed natural as native conformations are mainly stabilized by hydrophobic cores, and proteins can have a much higher ratio of core volume to total size than small peptides. (Note that many short peptides are known that are stabilized by disulfide bridges or metal ions; here we focus on peptides without such intra-chain bonds.)

Over the last years the traditional belief had to give way to a more differentiated picture: it was found that peptides can show a pronounced propensity for adopting a significant population of ordered secondary structure [3-6], and there have been examples of peptides with stable and well-defined tertiary structures [7] or [8]. Remarkably, although the latter peptides have native conformations, they are not just smaller versions of typically multi-functional proteins but either designed peptides (Trp-cage [8], 20 residues), or derived from single protein domains (Villin Headpiece [7], 36 residues), or they have no functions that require interaction with different specific molecules (antifreeze protein AFP I [9], 37 residues). Hence, it is plausible to assume that the observed rough correlation of larger size and well-defined conformation of proteins is not so much a consequence of elementary biophysics but of biological evolution in environments rich in specific functional interactions. Evolution may have selected molecules with numerous different and well-defined surface patches for specific functional interactions, and the necessity of harboring many such well-defined patches on the same molecule naturally leads to larger, conformationally stable polypeptides. Therefore, by ignoring such biological effects and concentrating on physical feasibility one can expect that it is possible to identify or design many more peptides that fold into stable native structures in the huge sequence space of 20 to 40 residues.

For biotechnology and medicine such peptides would be interesting tools [10,11]. In these disciplines it is often aspired to have a molecule with a single specific function, and this could be realized with molecules much smaller than a typical protein. Smaller molecules potentially also have better bioavailability, are less immunogenic and cheaper. An illustrative example is the design of small peptide mimotopes, i.e. peptides that mimic a large protein antigen by presenting just the epitope interacting with the antibody. In this case we require that the designed peptide is optimized with respect to several objectives simultaneously, e.g. small size, conformational stability, and presentation of the epitope in its native form and accessibility. The conventional way of solving such a problem is the definition of a scalar fitness function that combines these objectives, e.g. as a linear combination of single objectives. However, such combinations are notoriously problematic because the functional form of the fitness is usually unknown. A typical case is the use of experimental data (constraints from NMR experiments or X-diffraction data) and classical force field in the refinement of biomolecular structures. There, one usually combines experimental constraints and force field to a pseudo-energy. Often the functional form of this pseudo-energy is assumed to be a linear combination of the two terms with the weights determined from practical experience. If such a form is not known, the multitude of objectives cannot be combined to a single scalar objective function. A way to circumvent this problem is inferential structure determination [12,13] where simulation data is used to probabilistically rank possible solutions, though not to drive an optimization. Multi-objective- or Pareto-optimization could be applied there as an alternative. The fundamental difference between single-and multi-objective optimization is that the set of optima in general is much larger for the latter method as it allows for a flexible trade-off between the various objectives [14]. Practically, this means that during a single-objective optimization one often has only one optimum (which can be relatively arbitrary due to the choice of the objective function), whereas multi-objective methods usually forwards a whole set of optima, the Pareto-front (see Additional file 1). For the optimization of molecules Pareto-methods are currently addressed by several groups [15-17], though most of these researchers focus on the optimization of small molecules that do not adopt stable native conformations.

We have developed an algorithm for the optimization of realistic peptide models [18] of conformationally stable peptides. Briefly, the algorithm comprises the following five steps: (1) an optional step of transplanting a given epitope onto suitable carrier peptides of known 3D-structure; (2) automated modeling of several point mutants using as templates the energetically most favorable or otherwise fittest peptides of the parent generation; (3) the molecular dynamics (MD) of each point mutant in aqueous solution is simulated over a time of the order of 10 ns with a classical all-atom force field in explicit solvent; (4) extraction of fitness values from each trajectory; (5) selection of fittest individuals. The algorithm iterates over steps (2)-(5) an arbitrary number of times. To test algorithm and model we proposed the following computational experiment [18]: First a sequence of the well-characterized conformationally stable peptide Villin Headpiece (VH) [7] was perturbed by a point mutation that is expected to strongly destabilize the native VH structure by destroying its small hydrophobic core. If we subject this mutated peptide to our optimization algorithm with objective functions chosen to be compatible with the native structure (stability, native accessibility of some epitope loop), then the algorithm could recover the more stable native sequence of VH. In fact, the algorithm generated several mutants that, according to our computational results, should be even more stable than wild-type VH. As an example we have recently identified *in silico *the point mutant G34L [18].

Here we investigate how the algorithm explores the space spanned by amino acid sequences and the two objective functions global stability and accessibility of an epitope loop. To this end we analyze data from the sequence perturbation experiment introduced above, where we had performed two evolutionary optimization runs. Our results suggest that there could indeed be a considerable number of conformationally stable mutant peptides that may, in addition, fulfill other functionally related criteria. We present *in vitro *data on the structure and stability of the G34L mutant, where we find good agreement between computational prediction and experimental test.

## Results

### Evolutionary optimization

We have perturbed the sequence of VH by mutation F18K (note that we number residues in VH from 1 to 36, in deviation from the numbering scheme used in PDB entry 1vii [19], e.g. F18 in the present work corresponds to F58 in [19]). F18 stabilizes the native conformation, and even a relatively conservative mutation such as F18L destabilizes the fold [20]. We have introduced F18K, a much more drastic mutation that replaces the bulky hydrophobic side chain of Phe by the long, flexible, and positively charged side chain of Lys. Visual inspection of the wild-type 3D-structure [19] suggested that mutation F18K should strongly destabilize the native fold as it removes the phenyl ring of F18 from the small hydrophobic core of the peptide and replaces it by the positively charged side chain of Lys with no compensating negative charge nearby. In fact, in a MD simulation the mutant structure fell apart quickly.

Starting from F18K, the peptide sequence was then optimized with respect to two objective functions (see Eqs. 1 and 2 in Methods section): (1) the conformational stability *σ *of the peptide, (2) a quantity *α *related to the solvent accessible surface of an "epitope loop" comprising residues 12–17. These two objectives were chosen because they capture peptide properties that are often desired in practical applications. Specifically, maximization of conformational stability *σ *is important as it improves resistance against peptidases, and leads to well defined binding sites and thus lower entropical penalties on binding. In contrast to *σ*, which quantifies a global feature of a peptide, the objective function *α *describes a local aspect of the binding site, namely the negative root mean square deviation (RMSD) of residue-wise solvent accessible surfaces along the epitope loop between some target conformation and the peptide conformations generated in the course of the evolutionary optimization. (For *α *the *negative *RMSD value is used for convenience as it allows to treat the optimization of *α *as maximization, too.) Maximization of *α *is necessary to make sure that the binding face is exposed to a putative binding partner to the same extent as in a target conformation. Note that maximization of *α *will in general not be sufficient to drive the epitope loop towards some target conformation; this could be achieved by introducing a conformational RMSD of the epitope loop as third objective function. In this proof-of-concept study we have restricted the number of objective functions to two in order to facilitate data analysis.

Pareto-optimization should maximize both objective functions simultaneously towards their theoretical maximum values of *σ*_*max *_= 1 and *α*_*max *_= 0, respectively. This maximization is effected by using Pareto-dominance [14] as fitness criterion in the evolutionary optimization: The fitness values of non-dominated peptides are higher than those of dominated peptides. (A peptide *i *generated in the course of the optimization is said to strictly dominate a peptide *j *if *σ*(*i*) > *σ*(*j*) ^ *α*(*i*) > *α*(*j*).)

The two evolutionary optimization runs started from the same F18K mutant structure modeled *in silico *but with different random seeds. In each of the runs we produced 15 generations of peptides, each generation comprising 8 individual sequences and their respective structures. As evolutionary operation solely point mutations were applied to peptide sequences of the previous generation, so that the maximum number of sequence differences generated in the runs is 15 with respect to the initial F18K mutant, or 16 to the wild-type VH. Due to the costly fitness function the total computational expense was of the order of 1 year of CPU time.

**Figure 1:**
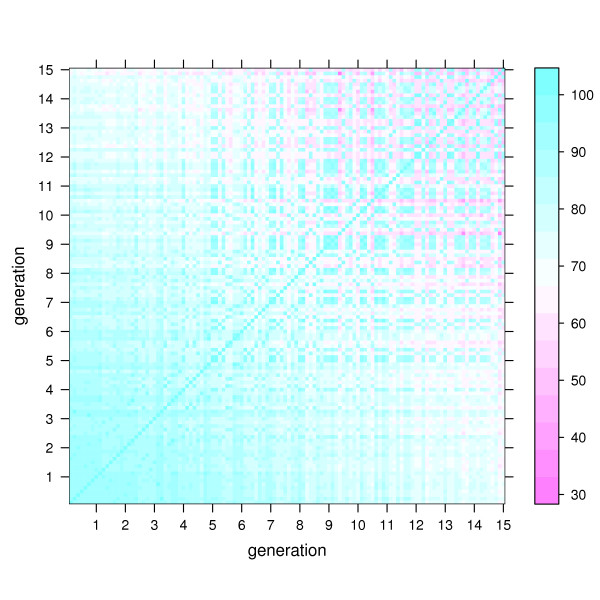
**Percentage identities *π*(*i*, *j*) of sequences in run 1**. All sequences *i *and *j *of individuals in run 1 are compared with respect to pairwise sequence identity. As there are 15 generations (15 bins along each axis of the plot), each with 8 individuals (8 stripes in each bin), there are 15·8 = 120 sequences and 120^2 ^comparisons, corresponding to the elements of the symmetric matrix in the figure. The legend at the right side of the plot shows the color coding of the *π *values (see also Methods section).

**Figure 2:**
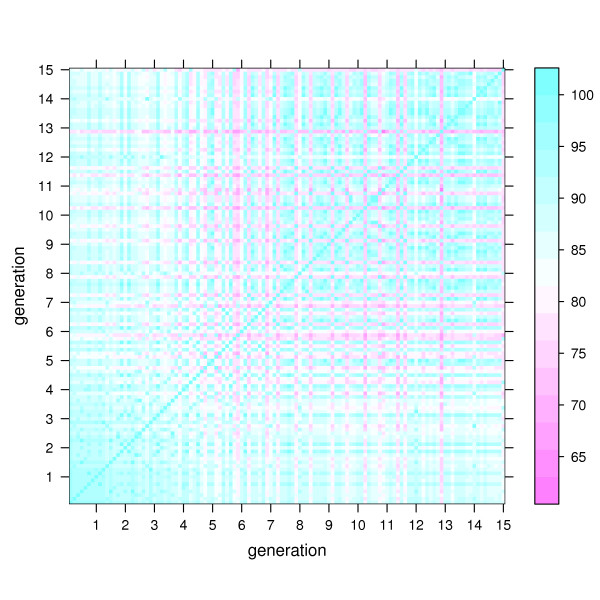
**Percentage identities of sequences in run 2**. Percentage identities of sequences in run 2. Analogous to Fig. 1, note however that the range of percentage identity values is narrower than in run 1.

The dynamics of the peptides in sequence space is visualized in Fig. 1 and Fig. 2 by showing the percentage *π*(*i, j*) of identities in optimal sequence pair alignments between any two sequences *i, j *generated in run 1 (Fig. 1) and 2 (Fig. 2). The maximum value of *π*(*i, j*) is 100 and means that both sequences are identical. The theoretical minimum value of 0 cannot be reached because of the conserved epitope loop (see also Methods section).

It can be seen in Fig. 1 and Fig. 2 that both runs start from a set of highly similar sequences. However, then the development becomes distinct between the runs.

In run 1 the population becomes slowly and steadily more diverse. In generation 15 at the end of the run *π *varies between about 40 and 100 within that generation (square at upper right corner of Fig. 1), which means that the sequences within that generation can differ at up to 22 sequence positions. With respect to the initial sequence, the sequences have *π *values of about 65 to 75 (square in lower right or upper left of Fig. 1), corresponding to about 13 to 9 mutations. Hence, the population seems to spread out from the initial sequence in different directions in sequence space.

In run 2 diversity first develops more quickly and culminates in generation 6. Towards the end of the run the intra-generation diversity decreases again to *π *values of above 90, with one outlier individual having a *π *of approximately 75 to the other individuals in generation 15. Remarkably, the intra-generation diversity at both start and end are low (lower left and upper right corners), while the difference between first and last generation are higher (lower right corner). This means that after 15 generations the whole population has settled in a region of sequence space different from the initial region. Overall, in the second run *p *remains in a more narrow range between about 65 and 100.

The evolution in sequence space described above is the result of point mutations and selections according to Pareto-dominance with respect to *σ *and *α*. Consequently, the Pareto-front defined by the non-dominated individuals shifts to the right to higher values of *α*, and upwards to the maximum *σ *= 1 in both runs (see also figures 5 and 6 in Additional file 1).

**Figure 3:**
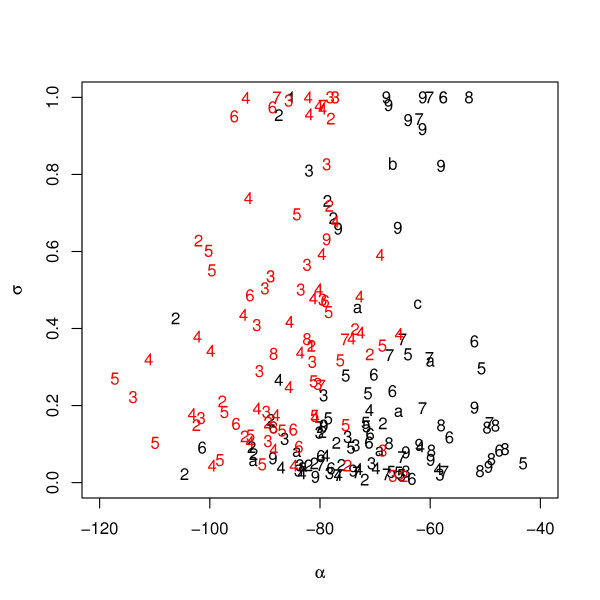
**Stabilities and accessibilities of all individuals**. Stability and accessibility values of all individuals from run 1 (black) and run 2 (red). Plot symbols indicate numbers of mutations of individuals with respect to wild-type VH (characters a, b, and c correspond to mutation numbers 10, 11, and 12, resp.). The maximum number of mutations is 12 in run 1 and 9 in run 2.

The data from objective functions and sequence space is summarized in Fig. 3. It displays (*α*, *σ*) values of all individuals from both runs and simultaneously, indicated by the plot symbols, the numbers of mutations with respect to the native VH sequence. The figure refers back to the initial hypothesis that there may exist many more peptides that fulfill certain criteria, including conformational stability. In fact, the figure shows that there are about a dozen sequences with 1 to 9 mutations and *σ *≈ 1, and that some of these also have *α *values of up to -50 (the runs actually generate 19 times individuals with *σ *= 1, but some of these are identical, so that there remain 12 different individuals).

Figure 3 has several other interesting features. The majority of individuals from both runs cluster at low *σ *values in the bottom part of the plot. The ranges of *α *values from both runs are wide and overlapping. This broad distribution is not astonishing for the following reason. We have computed *α *using the sampled conformation of lowest energy of the individual peptide, and for low values of the stability *σ *this single conformation (and hence its *α*) can be rather arbitrary. This means that for low *σ *values we must expect a noisy distribution of *α*.

The picture changes above about *σ *= 0.5. In a transition region of *σ *values between about 0.7 and 0.9, the density of individuals is reduced, and a gap opens between *α *values of -80 and -70. For high *σ *values the density increases again while the gap along the *α *axis persists, so that for maximum stabilities of *σ *≈ 1 we have two well-separated groups with *α *< -80 and *α *> -70, respectively.

The fact that in Fig. 3 there is an accumulation of individuals at high *σ *values seems to support our initial hypothesis. Note that it is not possible to conclude from the number of points in a certain region of the (*α*, *σ*) plane to numbers of individual peptides in that region, because sequences may have been sampled several times. However, the correction for this multi-sampling does not affect the overall picture (see Additional file 1 for details).

After the correction, there are nine sequences with a *σ *value of exactly 1, i.e. they form stable conformations according to the criterion described in the Methods section. These nine individuals are the most interesting members of the Pareto-front since they promise not only high stability but also well-defined structure and surface exposure of the epitope loop. Five of these individuals come from run 1 and in Fig. 3 are located right of the *α *gap (*α *> -70). Of the remaining four stable individuals, three are from run 2 and one is from run 1; all four individuals in Fig. 3 are left of the *α *gap (*α *< -75). We have compared these nine individuals with respect to sequence and structure, including in the comparison also the wild-type VH sequence and structure as tenth individual; all ten individuals are provided as supplementary material (Additional file 2).

**Figure 4:**
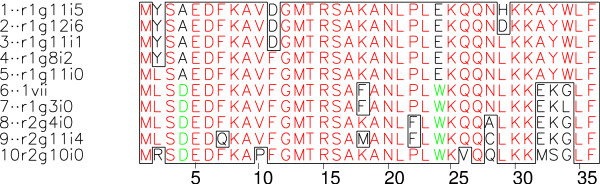
**Sequence alignment of stable individuals**. Multiple alignment of sequences of high-*σ *individuals from Fig. 5 (same numbering of sequences). The code r_*a*_g_*b*_i_*c *_on the left shows that sequence comes from run *a *∈ {1, 2}, generation *b *∈ {1, 2, ..., 15}, individual *c *∈ {0, 1, ..., 7}. 1vii is the wild-type sequence. Different colors in an alignment column are used to emphasize that the column is occupied by two different residues. Boxes separate residues that are in the minority in an alignment column from the majority. Figure prepared using Prettyplot [43].

All ten sequences are shown in a multiple sequence alignment in Fig. 4. The alignment shows that the sequences can be roughly grouped in two clusters, a non-native one (sequences 1–5), and a native one (sequences 6–10) that includes wildtype VH (sequence 6). The main features distinguishing non-native and native cluster cluster are A vs. D at position 4, E vs. W at position 24, and as largest difference a strictly conserved AYW motif at 32–34 in the non-native cluster that replaces a motif with consensus sequence EKG in the native cluster. Another notable feature concerns the initial perturbation K18: most of the sequences retain this K, especially all sequences in the non-native cluster.

**Figure 5:**
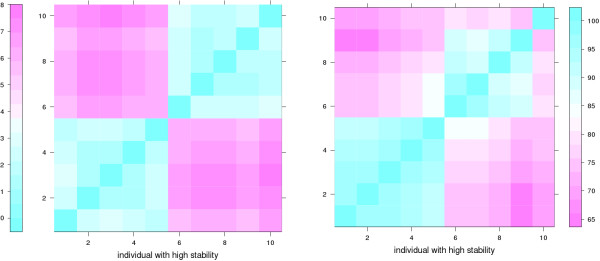
**Pairwise comparison of stable individuals**. Pairwise comparison of all sequences with highest stability (*σ *= 1) and wild-type VH (individual 6) with respect to structure (left) and sequence (right). The left panel shows the pairwise RMSD with respect to *C*_*α*_-positions after optimal superposition; color codes RMSD in units of 1 Å (= 0.1 nm). The right panel gives the percentage sequence identity between the same peptides as on the left (using same numbering of individuals as there). The figure shows that the individuals fall into two clusters or sub-folds, a cluster with modified fold and sequences (individuals 1–5) and a cluster similar to the wild-type (individuals 6–10).

Fig. 5 shows quantitatively by pairwise comparisons that the individuals not only cluster in sequence space, but also show the same clustering in terms of 3D-structure. The two clusters are clearly separated with *C*_*α *_RMSD values of below 3Å within each cluster and 6–8Å between the clusters. Overall, sequences follow an analogous pattern (right part of Fig. 5), though one of the individuals (number 10) from the native VH cluster has a sequence that seems to be roughly equidistant to both clusters in sequence space.

The two clusters correlate perfectly with the two groups left and right of the *α *gap, respectively, in Fig. 3, with the native cluster on the left and the non-native cluster on the right of the gap. It may be surprising that the *α*-values in the native-like cluster are lower than those in the non-native cluster. The reason for this is that the reference structure for the computation of *α *according to Eq. 1 is the experimental native structure [19] and not the native structure relaxed in force field simulations without experimental constraints. This relaxation leads to a shift of *α *in the native cluster to values below -75. Accordingly, the structures left of the *α *gap with *α *values around -80 have structures similar to the relaxed native VH structure.

**Figure 6:**
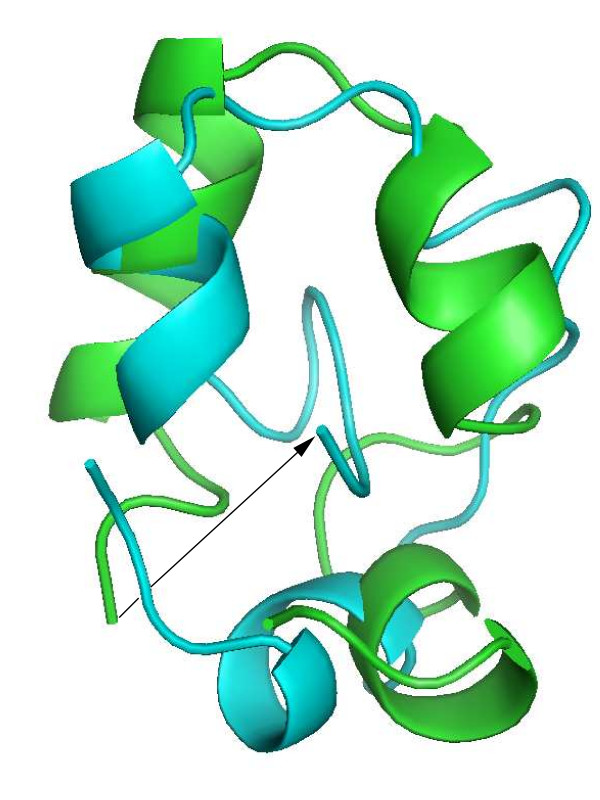
**Superposition of representatives from the two stable sub-folds**. Superposition of native VH (green) and sixfold mutant r1g11i0 (cyan). The two structures have been superimposed optimally with respect to their *C*_*α *_positions (RMSD 5.9 Å) using the Kabsch-algorithm [44]. The arrow marks the shift of the C-terminus.

The other cluster (individuals 1–5 in Fig. 4 and Fig. 5) is remarkable since it combines maximum *σ*, increased *α *values, and non-native structure. The superposition with the native VH fold in Fig. 6 shows that in the non-native cluster the N-terminal helix is tilted, the middle helix is almost dissolved into a number of turns, the C-terminal helix is shortened and tilted, and the C-terminal loop moves into the core. Nevertheless, as visible in Fig. 6, the global topology of the new fold is still related to that of the native VH. Hence, it may be appropriate to speak of the two clusters as of two sub-folds of the VH fold.

**Figure 7:**
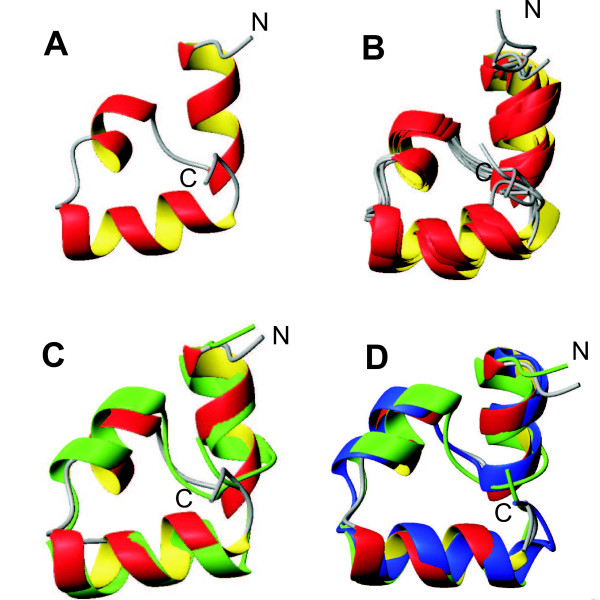
**Comparisons of experimental and predicted structures**. Experimentally solved solution NMR structure of the VH mutant G34L (deposited in PDB, code 2ppz). (A) Ribbon diagram of the lowest energy structure in terms of total energy. (B) Overlay of 5 final solution structures. (C) Comparison between the model structure (green) and the lowest energy experimental structure. (D) Comparison between the model structure (green), the wild-type X-ray structure (blue) and the lowest energy experimental structure.

All structures in the non-native cluster show the internalized C-terminal loop, the A_32_Y_33_W_34 _sequence feature mentioned above, and K18. In fact the internalized part mainly comprises this AYW motif and allows the formation of a hydrophobic cluster integrating also the aliphatic part of the sidechain of K18. The amino-tip of the latter is always exposed to the solvent, while in most of the non-native structures, the aliphatic part of K18 forms a broad contact with the aromatic ring of Y33 (see also Fig. 7 in Additional file 1).

**Figure 8:**
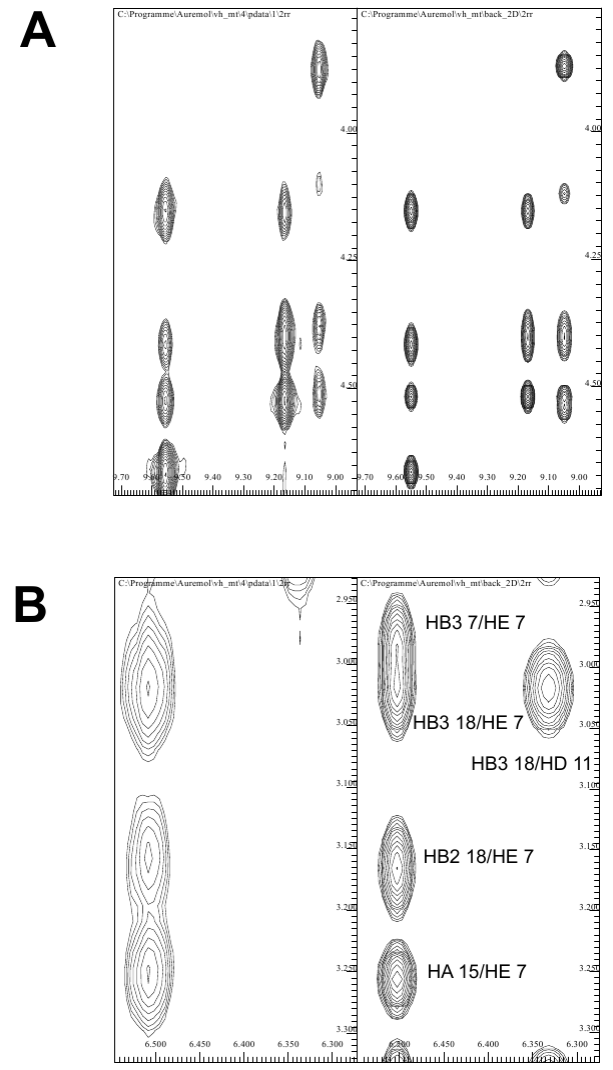
**Comparison of experimental and simulated spectra**. (A) Section corresponding to residues 3, 4, and 7 of the experimental 2D NOESY spectrum of the VH mutant G34L. Right: The corresponding region of the simulated spectrum. Simulations were performed using the predicted structure. (B) A different region is cut out of the whole spectrum. Again in general both regions agree well. However, the signal corresponding to HB3 18/HD 11 is visible in the simulated spectrum on the right side while it is completely missing in the experimental one, indicating for this region a different side-chain orientation between prediction and true solution structure.

Another structural rearrangement accompanying the integration of the C-terminal loop into the core is the move of the N-terminus (see Fig. 6). In the native fold, the position of the N-terminus may be stabilized by a salt-bridge between D4 and R15. The *C*_*β*_-distance lies at 4–5 Å in our models, and our experimental data (see below) supports that the locations of both sidechains allow a salt-bridge. As described above, we have in all non-native sequences at position 4 an Alanine so that a salt-bridge to R15 is no longer possible. This should facilitate large rearrangements of positions 4 and 15 without energetic penalties. This is consistent with the observed increase of the *C*_*β*_-distance between residues 4 and 15 to 15–18 Å in the non-native fold models (see also Fig. 8 in Additional file 1). After this large shift, the N-terminus forms part of clamp into which the C-terminal loop is embedded (Fig. 6).

The third sequence feature that distinguishes native and non-native individuals is W vs. E at position 24. The native W24 has been found experimentally to be important for interaction of VH with its natural binding partner F-actin [21]. Visual inspection of the native structure of VH or the models of its homologs gives no indications why the structure of VH alone should be stabilized by W24. On the other hand does inspection of our non-native models suggest that E24 could stabilize the short *α*-helix comprising residues 23–28 by providing a capping interaction [22] with the N-terminus of this helix. This helix is also present in the natively folded models, and the fact that we do not see native models with E24 may be due to the stochastic nature of the sampling procedure.

There are also two structures in the native cluster (individuals 8 and 10) that have retained K18. They have found a surprisingly simple solution of how to accommodate this residue without changing the fold. The sidechain of K18 is bent sharply so that this aliphatic turn roughly takes the place of the phenyl-ring of the native F18 in the hydrophobic core, while the amino group is again exposed at the surface (see also Fig. 8 in Additional file 1).

The first structure with *σ *= 1 that we have encountered in our study was indeed a revertant (individual number 7 in Fig. 4) that reintroduced the native F18; but even this individual differs from the wild-type VH by mutation G34L. It had been reported in Ref. [18] that the wild-type VH according to our stability criterion is quite stable but does not reach *σ *= 1. In contrast, individual 7 has *σ *= 1, and thus promised to have a higher stability while leaving the fold native. This prediction – native fold with higher stability – has prompted our first experimental test. Results of this test are given in the experimental section below.

### Experimental results: 3D-structure and stability

The first peptide with *σ *= 1 generated in the evolutionary optimization was the point mutant G34L of wild-type VH. Amongst all 39 single and double mutants only two (corresponding to 5%) had this stability. In Fig. 3 G34L can be easily identified since it is the only individual from run 1 in the group with *σ *= 1 and *α *< -75. In Fig. 4 and Fig. 5 it corresponds to individual number 7. According to the simulation the peptide belongs to the native VH sub-fold but has a higher stability than the native VH. To test these predictions we have carried out an experimental three-dimensional NMR structure determination together with denaturation experiments monitored by CD spectroscopy. Details are given below and in the experimental part of the Methods section.

For NMR structure determination, a total of 388 NOE distance restraints obtained from the 2D NOESY spectrum were used in the final structure calculations. These were distributed as 193 intra-residual restraints, 142 sequential restraints, 42 medium-range restraints (|*i *- *j*| ≤ 4), and 29 long-range restraints (|*i *- *j*| > 4). In addition, 24 Φ and 24 Ψ angle restraints were obtained from an analysis of the ^1^H secondary chemical shifts. Based on these experimental restraints, structures were calculated using a simulated annealing protocol in the program CNS [23] and the best structures in terms of total energy were selected for further analysis. In agreement with the prediction, the analysis of the experimentally solved structures shows that the molecule folds into one stable globular domain that consists of three *α*-helices. Helices H1, H2 and H3 comprise residues Asp4 to Phe11, Arg15 to Ala19, and Leu23 to Glu32, respectively. Fig. 7A shows the experimental structure possessing the lowest total energy in a ribbon representation. An overlay of five of the final solution structures is shown in Fig. 7B. In the set of ten selected final solution structures the average RMSD value to the mean structure amounts to 1.7 Å for the *C*_*α *_atoms. The experimental structure with the lowest total energy is compared in Fig. 7C with the predicted structure shown in green. The average pairwise RMSD value between the model structure and the ten selected final NMR structures is 2.9 Å. It is clear that both model and experimental NMR structure display the same global fold, while minor differences can be observed for the regular secondary structure elements; especially the second *α*-helix has a slightly different orientation in the model structure compared to the NMR structures.

One reliable way to test the agreement between experimental data and a three-dimensional structure is to back-calculate the experimental data from the trial structure and then to compare simulated and experimental data. We have been using for this purpose the AUREMOL module RELAX [24-26]. To check the agreement of our model structure with the experimental 2D NOESY spectrum, its spectrum was back-calculated based on the predicted structure of G34L and the experimental resonance line assignment. As an example two corresponding sections of these spectra are displayed in Fig. 8A with the experimental spectrum on the left and the simulated spectrum on the right. Both sections correspond to the N-terminal amino acids Ser3, Asp4, and Phe7. The two sections are virtually identical indicating that the model structure fits the experimental data very well for these residues. In general the comparison of the experimental and simulated data indicates similarly to the comparison of the corresponding structures that the predicted structure is fairly close to the experimental data. However, it should be noted that the results also indicate that the orientations of several side-chains such as Phe 11 differ between model and experimental data (Fig. 8B) for example in the simulated spectrum an interaction between HB3 18 and HD 11 is visible while this interaction is missing in the experimental spectrum. In this context, one has to consider that even the stable peptides with *σ *= 1 still can have some conformational flexibility, especially in the less well ordered loop regions, and what one observes in solution with NMR spectroscopy is an ensemble average. A single model structure as it was used for the data simulation can only represent one of many possible sub-states present in solution. Recently a high resolution X-ray structure of the wild-type VH has been published [27]. In Fig. 7D the predicted structure (green) is compared to both the X-ray structure (blue) and the best NMR structure in terms of total energy (red). Obviously, all three molecules adopt the same three-dimensional fold, and the limits of the regular secondary structure elements are almost identical.

**Figure 9:**
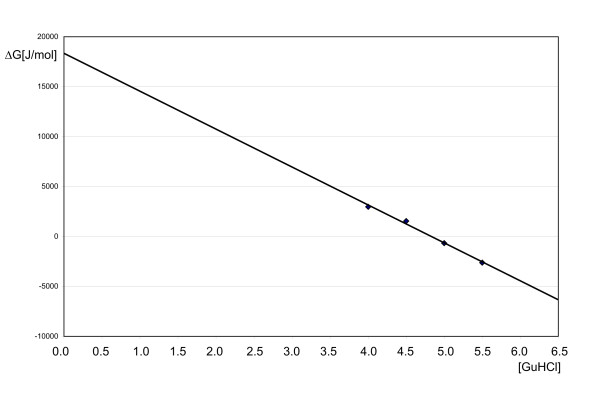
**Experimental stability**. GuHCl unfolding of the Villin Headpiece mutant G34L as monitored by CD spectroscopy. Shown is the linear extrapolation of the Δ*G *values determined in the transition region to 0 M GuHCl.

The stability of the peptide was experimentally determined from GuHCl denaturation experiments monitored with CD spectroscopy. Fig. 9 shows the linear extrapolation of the Δ*G *values determined in the transition region to 0 M GuHCl, giving a value of 18.2 kJ/mol. To allow a comparison with previously obtained data for the corresponding wild-type peptide, measurements were performed under similar conditions and also similar methods for data evaluation were used as described previously [7].

The experimentally determined Δ*G *value of 18.2 kJ/mol (4.3 kcal/mol) for the VH-mutant G34L is similar to the previously determined Δ*G *values of 3.3 and 4.1 kcal/mol obtained in H_2_O and D_2_O, respectively, for the wild-type peptide [7].

Our experimental data on structure and stability indicate that the *in silico *predictions are essentially correct with respect to the G34L mutant, namely that the structure of the peptide is not perturbed by this mutation but rather stabilized.

## Discussion

We have presented results on evolutionary *in silico *optimization of peptides with respect to two objective functions, including conformational stability. The optimization method is conceptually simple but computationally demanding due to the detailed modeling of the biomolecular system with classical all-atom force fields. This level of detail was chosen to allow for direct comparison with experiment, as shown above.

Despite the fact that classical force fields have proven their usefulness in numerous applications, they are not perfect. As we are performing extended molecular dynamics simulations, imperfections of the force field model may well influence conformational distributions [28]. Moreover, our simple single molecule model does neglect other factors that are important in experiments, such as the propensity to aggregate. Nevertheless, we think that the model is sufficiently peptide-like to allow conclusions from evolutionary optimization runs to real peptides.

With the high computational demands of the molecular model, efficiency is an important aspect. In view of the wide spread and seemingly erratic movements of objective function values (see Additional file 1) one may ask whether a simple random sampling of mutations may have led to similar results. We have tested this and found a maximum stability of 0.32 in such runs, i.e. with the same use of CPU time, random single mutations did not generate any stable individual (see Additional file 1). Given the large size of sequence space, this is not surprising and does not contradict our hypothesis that there exist many islands of stability. We think that these islands are probably surrounded by a much larger sea of instability. This picture was also one of the reasons why we have avoided more complex evolutionary operators such as cross-over that potentially permit hopping between islands. The other reason was that the larger changes brought about by these operators would be difficult to relax during molecular dynamics simulations of acceptable lengths. However, we are exploring other options, e.g. double mutations with their potential to mutually compensate deleterious effects on stability while changing other fitness terms of the molecule.

A point to consider is the danger of generating individuals that are kinetically trapped in meta-stable conformations. We think that the fact that we see stability in both native and non-native group, each comprising four to five individuals, tested for stability in as many independent simulations, backs the assertion that these conformations are not kinetically trapped. This has been further supported by several longer independent MD simulations in which the predicted structures have remained stable. Nevertheless, we will analyse their energy landscape further with suitable methods [29] and try to determine their structures *in vitro*.

The results evoke new questions, for instance whether the sub-folds are necessarily isolated or whether there may exist bridges between some of them. Optimization algorithms such as the presented one could also be applied to find peptides that switch between sub-folds, depending on temperature, pH, or other conditions.

## Conclusion

For the exploration of sequence-structure space of peptides we have used evolutionary Pareto-optimization, exploring sequence space with point mutations and evaluating proposed sequences with a computationally intensive, realistic model. A first *in vitro *test of computational results has supported the viability of the method. The application of the method to a perturbed sequence of the conformationally stable Villin Headpiece peptide generated two clearly distinguishable, stably folding clusters or sub-folds of peptides, each populated by several sequences: a native Villin Headpiece sub-fold, and a distorted sub-fold with a similar global topology. Since it is unlikely that Villin Headpiece is the only stable peptide that follows this pattern, we conclude that sub-folds could be a common fine-structure of the peptide fold-space, and that many undiscovered stably folding peptides may exist.

## Methods

### Evolutionary optimization

**Figure 10:**
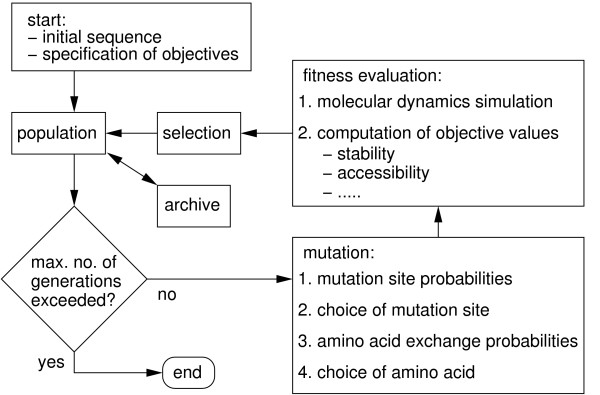
**Algorithm for evolutionary optimization**. Flow chart of evolutionary optimization used in present study.

For the *in silico *optimization, a multi-objective evolutionary algorithm has been used. The algorithm has been described in detail in [18]. Hence, we here repeat only the key elements of it (see Fig. 10). Point mutations were used as sole evolutionary operator to move through sequence space. Mutations were preferentially introduced at positions where they promised to be stabilizing; mutations were performed with Whatif [30]. To identify such positions we carried out computational alanine scanning for each new individual. At those positions where mutation to Ala led to the largest stabilization we introduced all possible amino acids and chose one of these randomly according to probabilities proportional to their predicted stabilizing effect; stabilization was computed by combining force field energies [31] and solvation energies [32]. The fitness of each new offspring individual was evaluated on the basis of a 10 ns molecular dynamics simulation with Gromacs 3.2 [31]. Individuals for the next generation were selected from the pool of the previous generation and its direct offsprings in a tournament using Pareto-dominance together with elitism [14,16] in the (*α*, *σ*) plane of objective functions (see below) as selection criterion. In more detail, in each tournament at first two individuals from the pool of parents and offsprings were chosen at random. Secondly, the two individuals were compared with respect to Pareto-dominance. If one of the individuals dominated the other individual the former was transferred to the next generation; if not, one of the two individuals was chosen at random. The first step, i.e. the random choice of a pair for the tournament is a mechanism to preserve diversity in the pool, while the second step prefers fitter individuals. Despite this preference, the random choice of pairs may lead to the extinction of the fittest individuals. In order to avoid this we used elitism, i.e. the best individuals generated during the optimization process were stored in an archive of fixed size to preserve individuals with top fitness; in each new generation, a fraction of these individuals was fed back from the archive.

Two objective functions were used, *α *and *σ*. The *α *value of an individual is defined as root mean square deviation (RMSD) solvent accessible surfaces (SAS) between residues on a target peptide and an individual peptide generated in the course of the optimization. This RMSD value is not computed for the whole peptide, but restricted to an "epitope" region:

(1) $$\alpha (i)=-\sqrt{\frac{1}{N_{ep}}\sum_{j=1}^{N_{ep}}{\left(SAS(i,j)-SAS(0,j)\right)}^{2}\frac{1}{{\text{Å}}^{2}}}$$

with the number *N*_*ep *_of residues in the arbitrarily selected epitope (here the six residues 12–17 with sequence GMTRSA); the sum running over all residues *j *in the epitope; *SAS*(*i, j*) the SAS (in Å ^2^) of residue *j *in the individual *i *generated in the course of the simulation; *SAS*(0*, j*) the corresponding surface of *j *in the target conformation (here: native wild-type conformation). SAS values were computed with naccess [33]. The maximum value of *α *is 0 in which case the epitope has the same residue-wise accessibilities in both the target and the individual conformation. The maximum value of *α *≈ -50 observed in the optimization runs corresponds to a difference between native and individual SAS of about the SAS of a single carbonyl oxygen atom.

The second objective function, *σ*, is defined as the fraction of conformations in a trajectory with a *C*_*α*_-RMSD to the initial conformation of that trajectory below a threshold *ρ*_*max*_:

(2) $$\sigma =\frac{1}{N_{steps}}\sum_{i=1}^{N_{steps}}\delta (\rho_{0,i}<\rho_{max})$$

with *N*_*steps *_the number of steps stored from a molecular dynamics trajectory; *ρ*_0,*i *_the *C*_*α*_-RMSD between the initial and the *i*th conformation stored from a trajectory; *ρ*_*max *_has been set to 3.5 Å as a result of previous analyses of molecular dynamics simulations of stable peptides; *δ *= 1 if the parenthesized argument is true and *δ *= 0 if it is false. If all recorded conformations in a molecular dynamics trajectory are close to the initial conformation, *σ *can reach its maximum value of 1, and the peptide can be considered conformationally stable.

The percentage *π*(*i, j*) of identities between two sequences *i, j *was computed from a multiple sequence alignment of all ten sequences shown in Fig. 4 with ClustalW [34]. For the progressive pairwise alignment step in ClustalW the following options were used: Needleman-Wunsch algorithm, Gonnet matrix, gap opening and extension penalties of 10 and 0.1, respectively. *π *values refer to the full sequence length of 36. Since the epitope loop 12–17 is not mutated, *p *values shown in Fig. 1 and Fig. 2 have a constant baseline at 16.7.

Most analyses in the computational part have been carried out with R [35], including the bio3d package [36]. Figures showing molecular structures were prepared using PyMOL [37].

### Peptide synthesis

The VH mutant G34L was synthesized on a MultiSynTec SYRO peptide synthesizer.

### NMR spectroscopy

For the NMR measurements 4.3 mg of the peptide were dissolved in 0.5 ml of 90%/10% (v/v) H_2_O/D_2_O and 0.1 mM 2,2-dimethyl-2-silapentane sulfonic acid (DSS) for internal referencing. The pH was adjusted to 5.0. NMR experiments were carried out at 298 K on a BRUKER DRX 500 MHz spectrometer equipped with three channels and a pulsed-field gradient triple-resonance probe equipped with *z*-gradients. The sequence-specific ^1^H resonance line assignment was performed using 2D TOCSY and NOESY spectra employing mixing times of 80 and 250 ms, respectively. NOE distance restraints were obtained from 2D NOESY spectra measured in H_2_O using a mixing time of 250 ms. Processing of the 2D data sets was accomplished using the Bruker software XWINMR while automated and manual spectra analysis was performed employing the software package AUREMOL [38].

### Structure calculations

Structure calculations were performed using the program CNS 1.1 [23] and a dynamic simulated annealing protocol for extended-strand starting structures. The Φ and Ψ angle restraints were obtained from an analysis of the proton chemical shifts using the program TALOS 2003.027.13.05 [39]. Accurate distance constraints were obtained from the NOE spectra by using the full relaxation matrix approach embedded in the AUREMOL module RELAX/REFINE (to be published). Appropriate individual error bounds were obtained by a local analysis of noise levels and signal overlap. In total 200 structures were calculated of which the 10 best in terms of total energy were selected for further analysis.

### Structure analysis

Secondary structure elements and RMSD values were calculated with the program MOLMOL 2K.2 [40] and structures were further analysed with PROCHECK v.3.4.4 [41].

### CD spectroscopy

Circular dichroism spectra were measured in an AVIV 62A-DS spectropolarimeter. For CD spectroscopy 20 *μ*g peptide was dissolved in 150 mM NaCl, 25 mM acetate and varying amounts of GuHCl at pH 5.0. For the guanidine hydrochloride (GuHCl) denaturation experiments 15 samples were prepared containing 0.0, 1.0, 1.5, 2.0, 2.5, 3.0, 3.5, 4.0, 4.5, 5.0, 5.5, 6.0, 6.5, 7.0, and 8.0 M GuHCl, respectively. Samples were equilibrated for 12 hours at 4°C. CD data were collected at 222 nm in a 5 mm path-length cell. For each step the average of 6 data points measured at an interval of 2 s was calculated. Data were baseline corrected by subtraction of a buffer-only experiment. The Δ*G *value at 0 M GuHCl was obtained by linear extrapolation of the Δ*G *values determined in the transition region [42].

## Authors' contributions

WG has designed, carried out and analysed NMR and CD experiments and written the experimental part of the manuscript; TH has implemented the evolutionary algorithm and carried out the simulations; DH has designed the computational experiments, analysed the computational results and written the main part of the manuscript. All authors read and approved the final manuscript.

## Supplementary Material

Additional file 1 This file contains several supplementary figures and descriptions. Click here for file

Additional file 2 We provide the predicted 3D-structures of the most stably folding individuals (sequences and names given in Fig. 4) as a zip-archive of PDB format files. The experimental structure of the wildtype (1-vii) is also included. All structures are superimposed so that the *C*_*α*_-RMSD to the wildtype structure is minimized. Click here for file
