# Supplementary material for: Evolutionary Pareto-optimization of stably folding peptides
Source: BMC Bioinformatics. 2008 Feb 19;9:109. doi: 10.1186/1471-2105-9-109 (PMC2263021; doi:10.1186/1471-2105-9-109)
Supplement: Additional file 1 — This file contains several supplementary figures and descriptions. [file 1471-2105-9-109-S1.pdf]

# Supplementary Material to "Evolutionary Pareto-optimization of stably folding peptides"

Wolfram Gronwald[1], Tim Hohm[2] , Daniel Hoffmann[*3]

[1]Institute for Functional Genomics, University of Regensburg, Josef-Engert-Strasse 9, 93053 Regensburg, Germany
[2]Computer Engineering and Networks Laboratory, ETH Zurich, Gloriastrasse 35, Zürich, Switzerland
[3]Centre for Medical Biotechnology, University of Duisburg-Essen, Universitätsstrasse, 45117 Essen, Germany

Email: Wolfram Gronwald - wolfram.gronwald@klinik.uni-regensburg.de; Tim Hohm - tim.hohm@tik.ee.ethz.ch; Daniel Hoffmann*- daniel.hoffmann@uni-due.de;

*Corresponding author

## Some terms in Pareto-optimization

Most real-world optimization problems require that a set of different and often incomparable objectives is considered. In conventional optimization (single-objective optimization) one tries to aggregate in an *ad hoc* manner the different objectives into a single objective. In contrast, Pareto- or multi-objective optimization techniques allow to optimize such a set of objectives simultaneously without the necessity of aggregating unrelated objectives in a single function.
Multi-objective optimization aims at simultaneously optimizing $m$ objectives $F = (f_1, \ldots, f_m) : \mathbb{R}^n \rightarrow \mathbb{R}^m$ depending on a decision vector of $n$ parameters or decision variables $x = (x_1, \ldots, x_n)$. These parameters may have to fulfill a set of $k$ constraints $g_i(x) \geq 0 \quad \forall i \in \{1, \ldots, k\}$. Without loss of generality it can be assumed that all objectives are to be minimized.
Since often a set of incomparable objectives is considered, one has to clarify the notion of "optimal solutions". A key term in this respect is Pareto-dominance:

*Pareto-dominance*

Given two objective vectors $x'$ and $x''$ it is said that $x'$ strictly dominates $x''$ (denoted $x' \succ x''$) if the following is true:

$$f_i(x') \leq f_i(x'') \, \forall i \in \{1, \dots, m\} \ ,$$

$$F(x') \neq F(x'') \ .$$

In terms of the Pareto-dominance the set of best solutions for a given multi-objective optimization problem covers all Pareto-optimal solutions and is called Pareto-set or Pareto-front.

*Pareto-optimality and Pareto-set*

A decision vector $x^*$ is called Pareto-optimal if

$$\nexists \, x \ \in \ X \, with \, x \succ x^*.$$

The set $X^*$ of all Pareto-optimal solutions, which is a subset to the set $X$ of all feasible solutions, is called Pareto-set

$$X^* = \{x^* \ \in \ X | \nexists \, x \ \in \ X : x \succ x^*\}.$$

Multi-objective optimization approaches aim at identifying a good approximation of the Pareto-set for a given problem. This approximation is made up from all non-dominated solutions found during the optimization process.

*Non-dominated solution*

In a given set of solutions represented by vectors of decision variables, an individual $x_{non-dom}$ is *non-dominated* if no other member of the set dominates $x_{non-dom}$ in terms of the Pareto-dominance relation.

A comprehensive treatment of Pareto-optimization can be found in the book by Deb (see references of main text).

**Correction for multiple sampling of sequences in Fig. 3 of main text**

We cannot conclude simply from the number of points in a certain region of the $(\alpha, \sigma)$ plane (Fig. 3 of main text) to numbers of individual peptides in that region. To understand this it is necessary to recapitulate that in our algorithm each offspring peptide in sequence space differs

from its parent by a single point mutation and inherits the parental “native” conformation. The offspring can then adapt its native conformation to the new sequence in a molecular dynamics simulation. Hence, in general we will see a change of native conformations concurrent to the evolutionary trajectories in sequences space. This means that if a sequence is revisited during this development, it will in general inherit a different native state from its parent and thus possibly produce different values of $\sigma$ and $\alpha$. In fact, we have observed several times that a sequence is sampled twice: first, it has a low $\sigma$ since during the 10 ns molecular dynamics simulation it often is not able to relax quickly enough from an unsuitable conformation into a stable native conformation (if the latter is available at all); then, a few generations later the same sequence is confronted with a conformation that is more native-like and then generates high $\sigma$ values. For a histogram of $\sigma$ frequencies (see Fig. 4) we have therefore corrected the data shown in Fig. 3 of the main article by counting each sequence only once and with its highest $\sigma$ value. This correction does not change the overall features of the $\sigma$ distribution seen above, i.e. the main contribution at low $\sigma$, the sparsely populated transition region between about 70 and 90, and the second peak at $\sigma \approx 1$. Although in Fig. 4 the two runs differ in detail they both share these gross features, including the peak at $\sigma \approx 1$.

### Random sampling

It is valuable to test the optimization algorithm against a random sampling for two related reasons. Firstly, if individuals with high stability and accessibility were very frequent in sequence space, it would not be necessary to use optimization algorithms to identify such individuals. Secondly, if an optimization algorithms yielded optimal sets of a similar quality than sets from random sampling runs, the optimization algorithm could not be called efficient. Therefore we have carried out a random sampling run that generated 120 individuals, i.e. the same number of individuals as in each of the optimization runs. Sequences were generated by random single point mutations. No sequence was sampled twice. The objective functions $\alpha$ and $\sigma$ were computed as described in the main text for the optimization algorithm.

Fig. 10 shows the comparison of the distribution of individuals from both optimization runs (black and red) and from random sampling (green). The cloud of randomly sampled individuals has its maximum density close to $\sigma = 0$ with the most stable outlier at $\sigma = 0.32$. In $\alpha$-direction the extension of the random cloud is much broader and even reaches values of up to -32, which

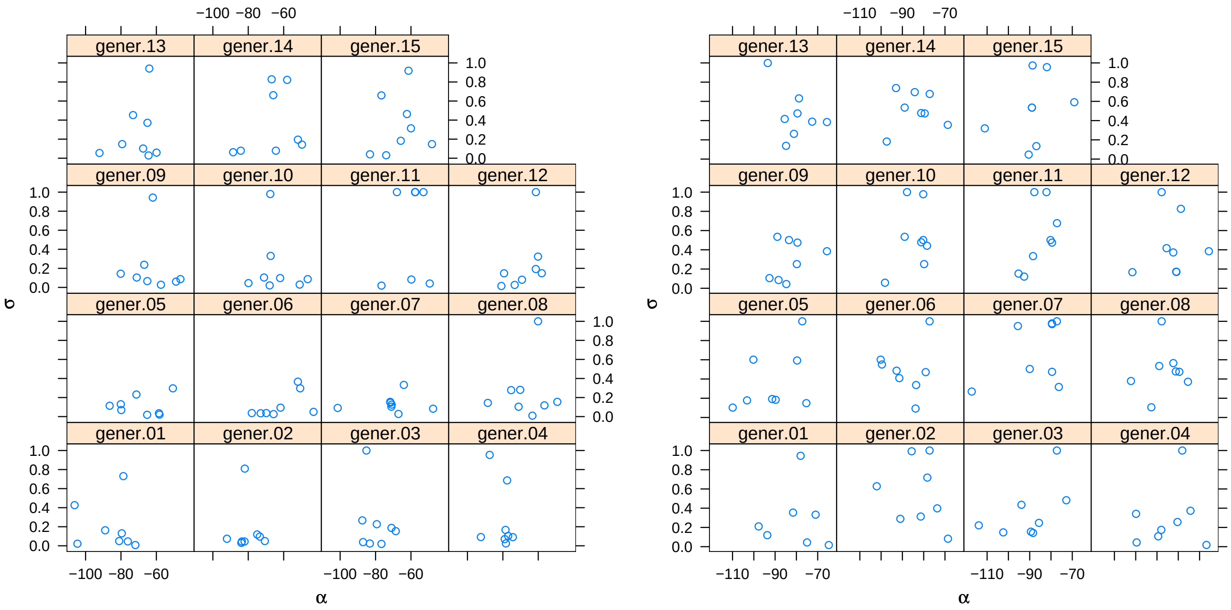


Figure 1: Evolution of populations over 15 generations each with 8 individuals in the space spanned by the two objective functions loop accessibility $\alpha$ and conformational stability $\sigma$ (for definitions see Methods section). The left and right part of the figure correspond to run 1 and 2, respectively. Accessibility scales differ between runs.

lies above those from the optimization runs. However, as we have explained in the main text, $\alpha$ values of very unstable individuals (low $\sigma$) can be rather arbitrary and are therefore not significant. The set of individuals generated by the optimization algorithm markedly differs from the random set, with the upper two thirds of the plot being exclusively populated by individuals from the optimization runs.

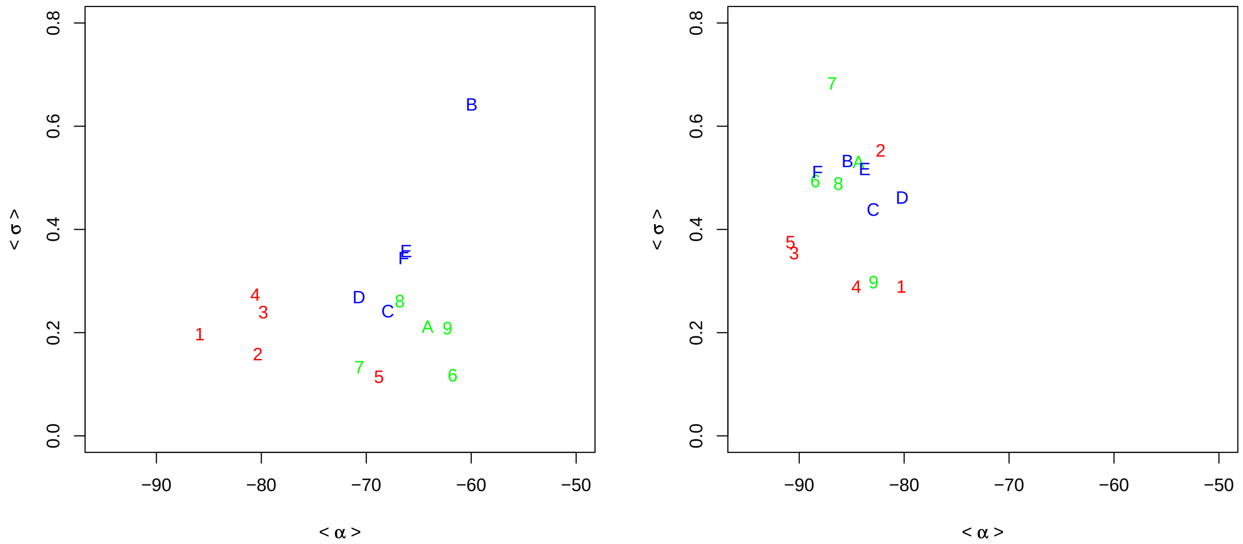


Figure 2: Evolution of the population centers from Fig. 1 for run 1 (left) and 2 (right) in the plane spanned by the generationwise averages $\langle\alpha\rangle$ and $\langle\sigma\rangle$ of the objective functions. The population centers in each generation are marked by generation numbers for generations 1-9 and by letters A-F for generations 10-15. An overall trend to fitter individuals can be seen as shift of the cloud of centers from the initial five generations (red), over the next five generations (green), to the final five generations (blue).

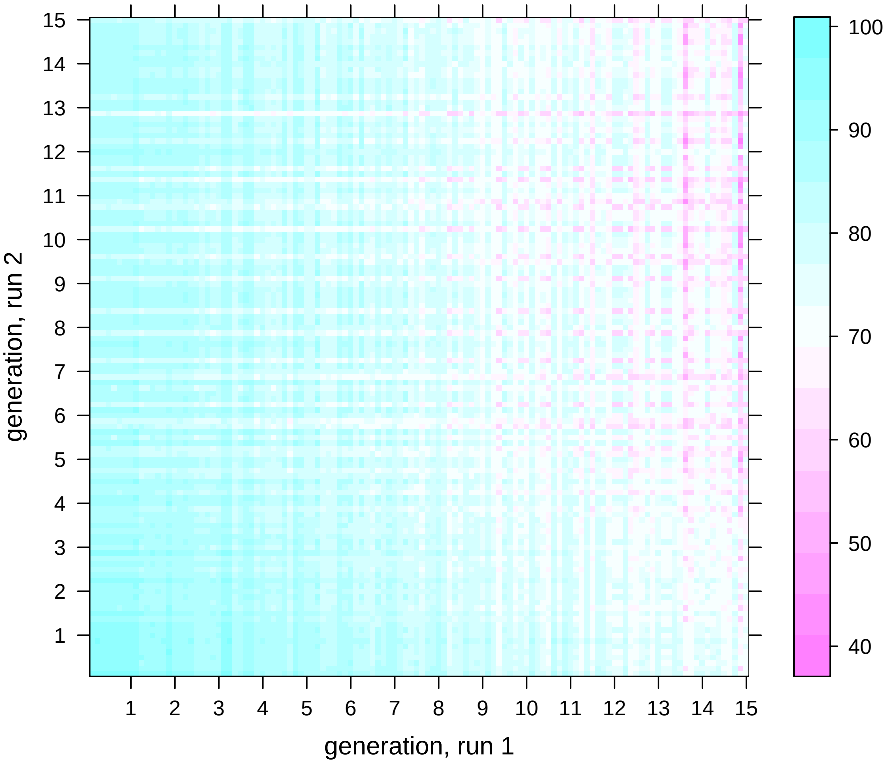


Figure 3: Percentage identities between runs 1 and 2. In contrast to Figs. 3 and 4 of the main article each of the two axes refers to a different run and the matrix is not symmetric. The range of percentage identity values differs from Figures 3 and 4.

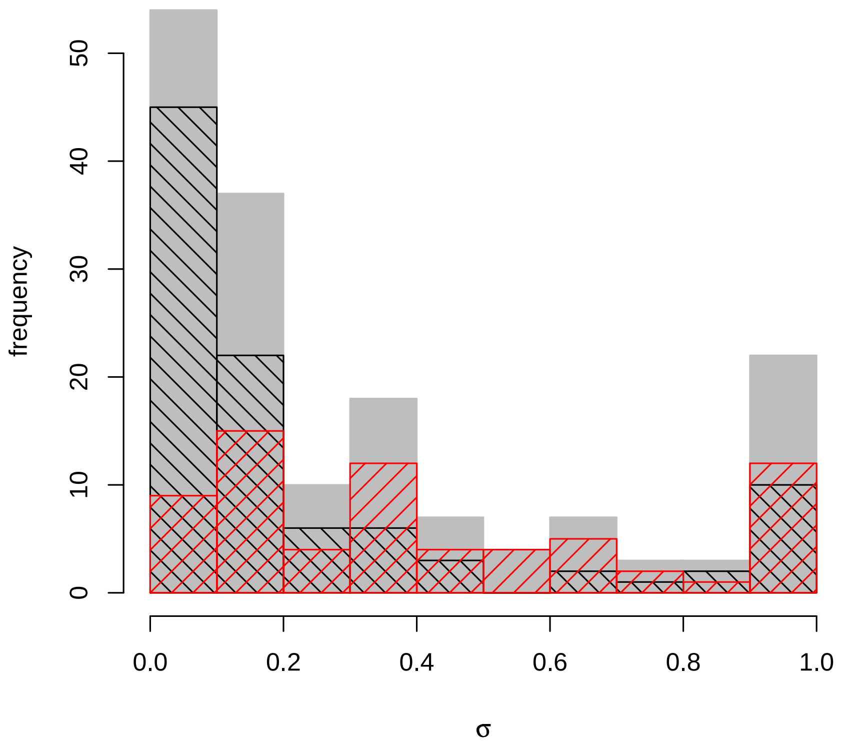


Figure 4: Histograms of stability $\sigma$ in run 1 (black), run 2 (red), and the sum of both runs (grey). As described in the main text, the data is corrected by considering each sampled sequence only once in each run even if it is sampled more frequently. This leads to different contributions from the two runs.

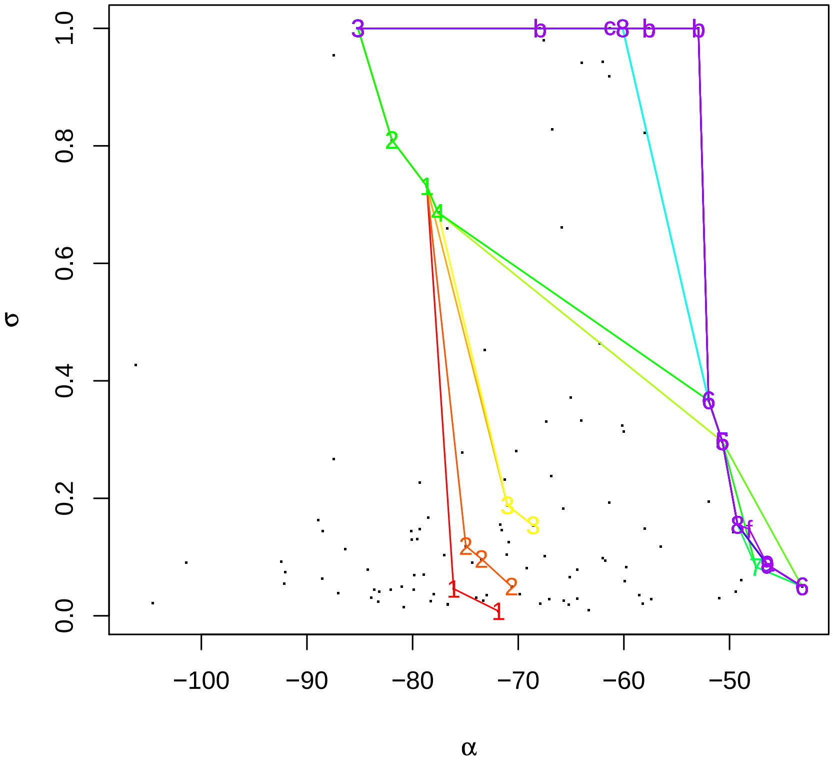


Figure 5: Development of the Pareto-front in run 1. $(\sigma, \alpha)$-pairs of all individual peptides are shown as black dots. Numbers mark individuals at the Pareto-front that have been sampled for the first time in the generation indicated by the respective number or letter $(1, 2, \ldots, e, f)$. Colored lines connecting these points give a rough impression of the Pareto-front. Coloring is according to the rainbow and encodes generation from red (generation 1) to violet (generation 15).

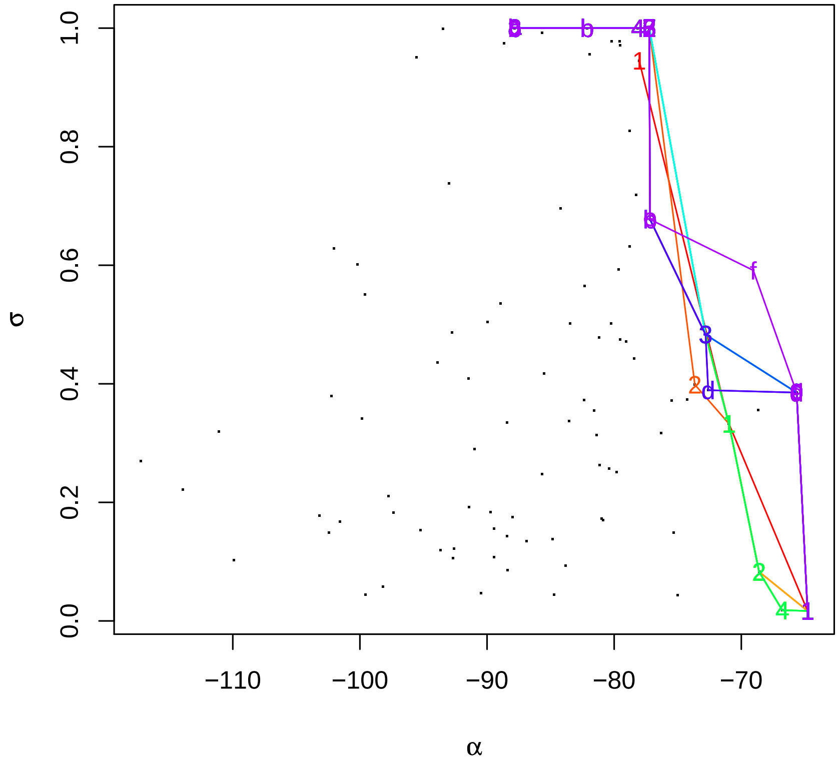


Figure 6: Development of the Pareto-front in run 2 (see also Fig. 5).

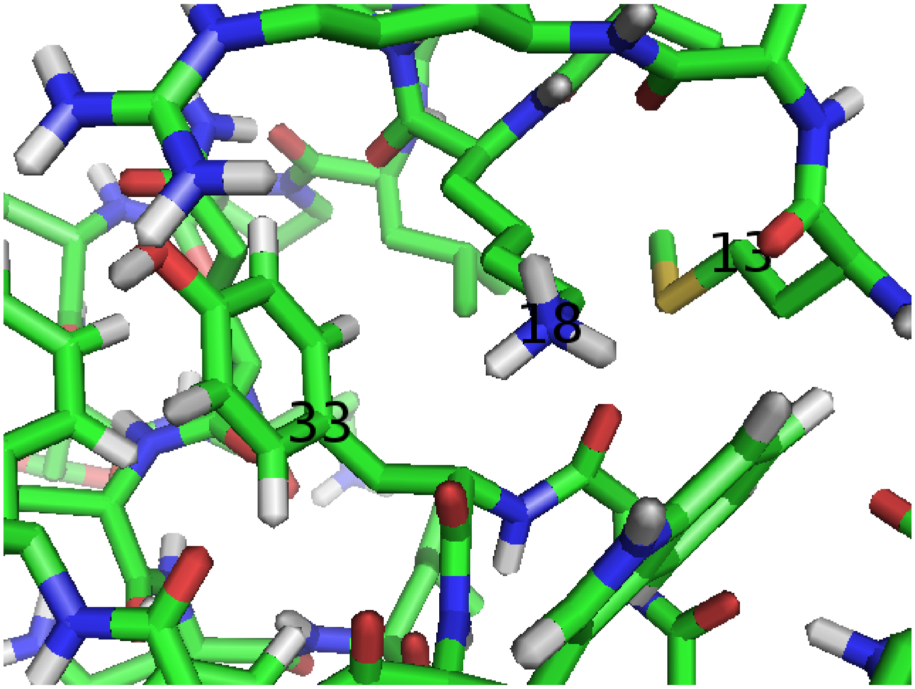


Figure 7: Close-up of typical structure (individual 3) from non-native cluster around K18. The aliphatic part of the K18 sidechain is integrated into the hydrophobic core, e.g. by contacts with the phenyl-ring of Y33 and the sidechain of M13. The amino-tip of K18 is exposed to the solvent. Note also that several carbonyl-oxygens are pointing towards the amino-group.

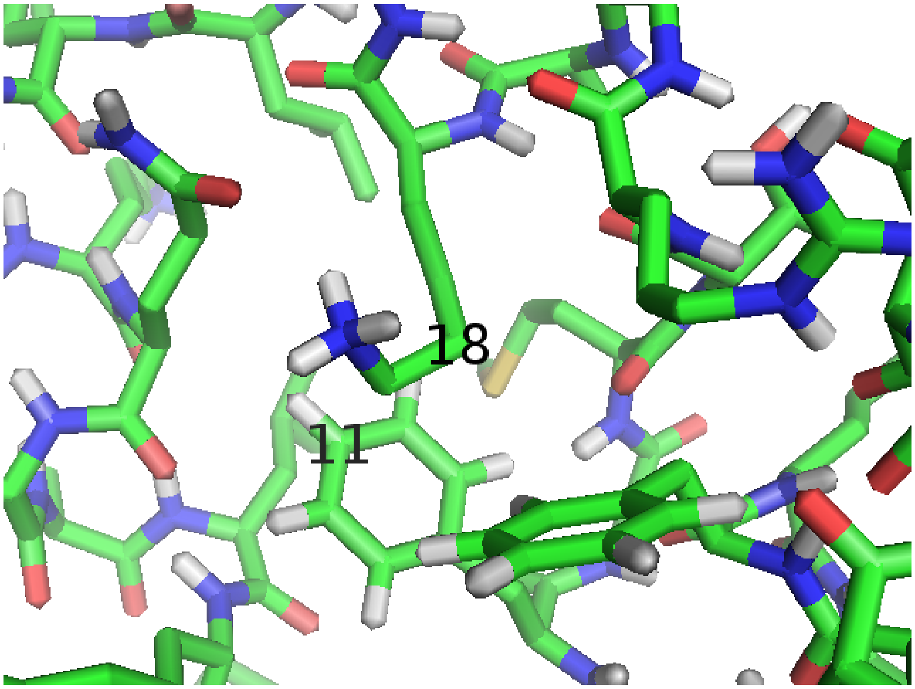


Figure 8: Close-up of structure (individual 8) from native cluster around K18. The aliphatic part of K18 is sharply bent, and this turn is included into the hydrophobic core, e.g. by contact with F11 underneath. Again, the amino-tip of K18 is exposed to the solvent.

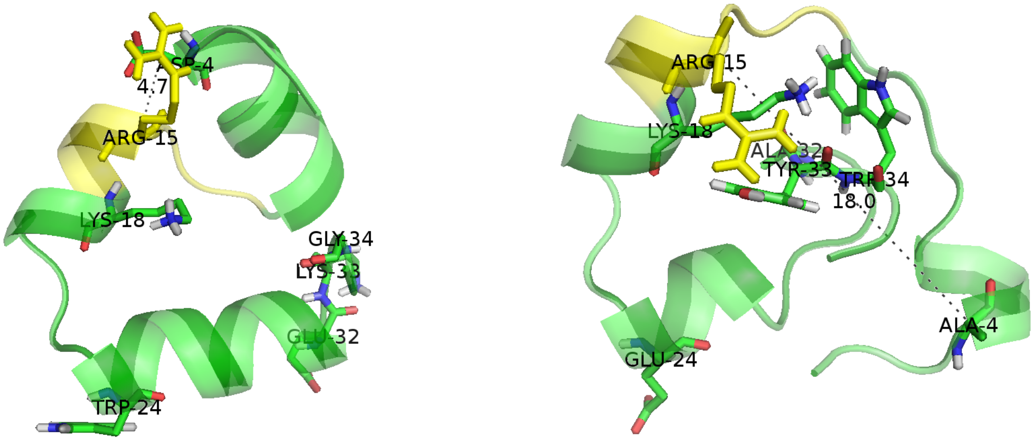


Figure 9: Overview of changes between native (left, individual 8) and non-native folds (right, individual 3). Key motifs that distinguish native from non-native sequences are labelled. The epitope loop is drawn in yellow. Also shown are the distances of $C_\beta$ 4 and 15.

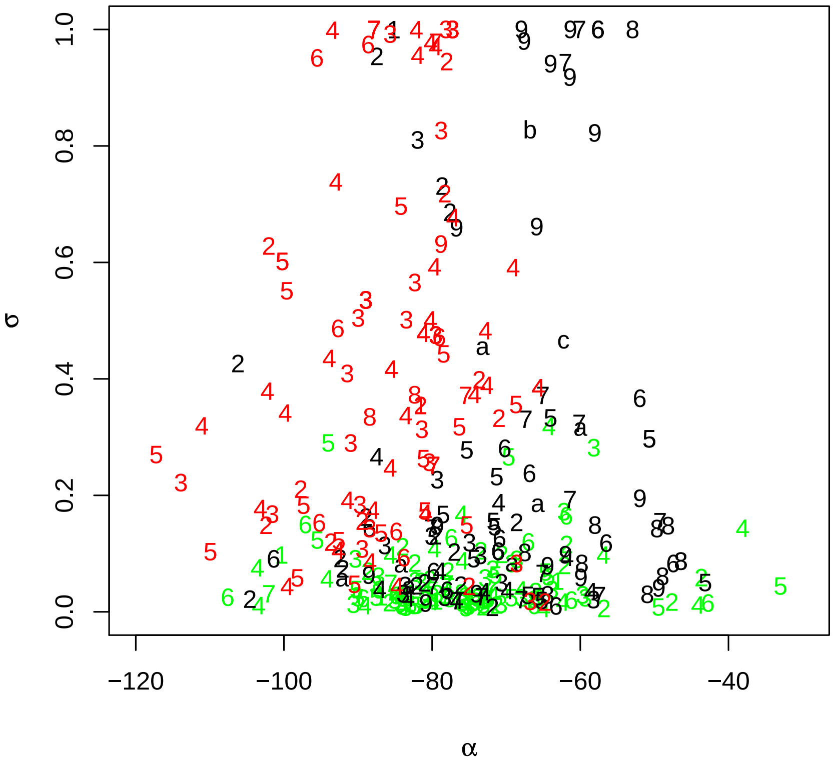


Figure 10: Distribution of individuals from both optimization runs (black, red) and random sampling (green). Numbers are mutations with respect to wild type VH (see also Fig. 3 in main text).
